# Novel mechanism of harmaline on inducing G2/M cell cycle arrest and apoptosis by up-regulating Fas/FasL in SGC-7901 cells

**DOI:** 10.1038/srep18613

**Published:** 2015-12-18

**Authors:** Yihai Wang, Chunhua Wang, Chenguang Jiang, Hong Zeng, Xiangjiu He

**Affiliations:** 1 School of Pharmacy, Guangdong Pharmaceutical University, Guangzhou 510006, China; 2 Huangshan Jingzhi Pharmaceutical Company of Nanjing Tongrentang Group, Huangshan 245999, China; 3 Xinjiang Production & Construction Corps Key Laboratory of Protection and Utilization of Biological Resources in Tarim Basin, Tarim 843300, China

## Abstract

Harmaline (HAR), a natural occurrence β-carboline alkaloid, was isolated from the seeds of *Peganum harmala* and exhibited potent antitumor effect. In this study, the anti-gastric tumor effects of HAR were firstly investigated *in vitro* and *in vivo*. The results strongly showed that HAR could inhibit tumor cell proliferation and induce G2/M cell cycle arrest accompanied by an increase in apoptotic cell death in SGC-7901 cancer cells. HAR could up-regulate the expressions of cell cycle-related proteins of p-Cdc2, p21, p-p53, Cyclin B and down-regulate the expression of p-Cdc25C. In addition, HAR could up-regulate the expressions of Fas/FasL, activated Caspase-8 and Caspase-3. Moreover, blocking Fas/FasL signaling could markedly inhibit the apoptosis caused by HAR, suggesting that Fas/FasL mediated pathways were involved in HAR-induced apoptosis. Interestingly, HAR could also exert on antitumor activity with a dose of 15 mg/kg/day *in vivo*, which was also related with cell cycle arrest. These new findings provided a framework for further exploration of HAR which possess the potential antitumor activity by inducing cell cycle arrest and apoptosis.

Harmal (*Peganum harmala* L.) is a perennial, glabrous plant which grows spontaneously in semi-arid conditions, steppe areas and sandy soils, native to eastern Mediterranean region. It is also found in the eastern Iranian region, North Africa, Middle East, China and some regions of the western USA^1^.

*P. harmala* is one of the few plants which are extensive and carefully used for traditional treatment of different diseases. Its seed is regarded as traditional therapeutic for asthma, diarrhea, diabetes, hypertension, jaundice, lumbago, maceration or infusion for fever, subcutaneous tumors^2^,^3^.

The seeds of *P. harmala* contain a variety of hallucinogenic alkaloids, such as harmine, harmane, harmalol, harmol, harmaline (HAR) and harmalidine, etc. These alkaloids are classified as β-carboline alkaloids according to their structural backbone. The β-carboline alkaloids have recently drawn attention due to their antitumor activities. Further mechanistic studies indicate that β-carboline derivatives inhibit DNA topoisomerases and interfere with DNA synthesis. Moreover, some β-carboline alkaloids are specific inhibitors of cyclin dependent kinases (CDKs)^4^. Among other β-carboline derivatives, harmaline (Fig. 1) acquires a prominent place, and is widely studied over the decades for its pharmacological effects, including anti-leishmanial^5^, anti-microbial^6^,^7^, anti-plasmodial^8^, anti-tumor^9^,^10^,^11^,^12^,^13^, hypothermic and vasorelaxant activities^14^.

The eukaryotic cell cycle progression is regulated by cyclin-dependent kinases (CDKs) and Cyclins. Progression through G1 into S phase is regulated by the Cyclin A-Cdk2 and Cyclin E-Cdk2 complexes, while Cyclin B-Cdc2 plays a role in G2/M phase transition. Cip/p21 is a universal inhibitor of CDKs that also regulates the G2/M phase transitions by binding to CDK-Cyclin complex and inhibits its kinase activity. Also p21 comes into the spotlight as a mediator of p53 tumor suppressor activity. Cdc25C is another critical regulator of Cdc2-Cyclin B1 kinase activity and control cell cycle progression by dephosphorylating and activating CDKs^15^.

The cell death receptor Fas is a major mediator of the apoptotic pathway. Upon stimulated by its ligand, FasL, the death receptor oligomerizes, recruits different adaptor proteins which activate the initiator caspase-8 and caspase-3 to induce cell apoptosis^16^.

In the present study, the detail antitumor mechanism of HAR in gastric tumor cells (SGC-7901) was firstly investigated. The results suggested that HAR abrogated tumor cell proliferation through inducing G2/M cell cycle arrest by increasing cyclin-dependent kinase inhibitor (CDKI)-p21 levels and modulating the activity of Cdc25C/Cdc2 *in vitro* and *in vivo*. Further, HAR could inhibit SGC-7901 cell proliferation by activation of Fas/FasL and two key downstream effectors (Caspase-8 and Caspase-3) through apoptosis pathway.

## Results

### Structure identification of HAR

The purified compound from *P. harmala* showed a positive response with Drangendorff’s reagent. The chemical structure was determined by its ^1^H- and ^13^C-NMR, as well as mass spectrum. The ^1^H-NMR spectrum exhibited the signals of three aromatic protons at *δ*_H_ 7.41 (d, *J* = 8.7 Hz, 5-H), 6.86 (d, *J* = 2.0 Hz, 8-H), and 6.70 (dd, *J* = 8.7, 2.0 Hz, 6-H), one methyl group at *δ*_H_ 2.25 (3H, s, –CH_3_), two methylene groups at *δ*_H_ 3.64 (2H, 3-H), 2.68 (2H, 4-H), one methoxyl group at *δ*_H_ 3.79 (3H, s, CH_3_O–) and an active hydrogen at *δ*_H_ 11.19 (1H, N-H). The ^13^C-NMR spectrum exhibited signals for 13 carbons at *δ*_C_ 19.2 (C-4), 22.0 (–CH_3_), 47.6 (C-3), 55.2 (CH_3_O–), 94.6 (C-8), 110.3 (C-6), 114.6 (C-5α), 119.4 (C-4α), 120.4 (C-5), 128.5 (C-9α), 137.6 (C-8α), 156.8 (C-1), and 157.1 (C-7), respectively. The spectra data were in accordance with that published in the previous literature^17^. Therefore, the structure of the purified compound was identified as harmaline (HAR).

### HAR inhibited the proliferation of SGC-7901 cells

**Figure 2:**
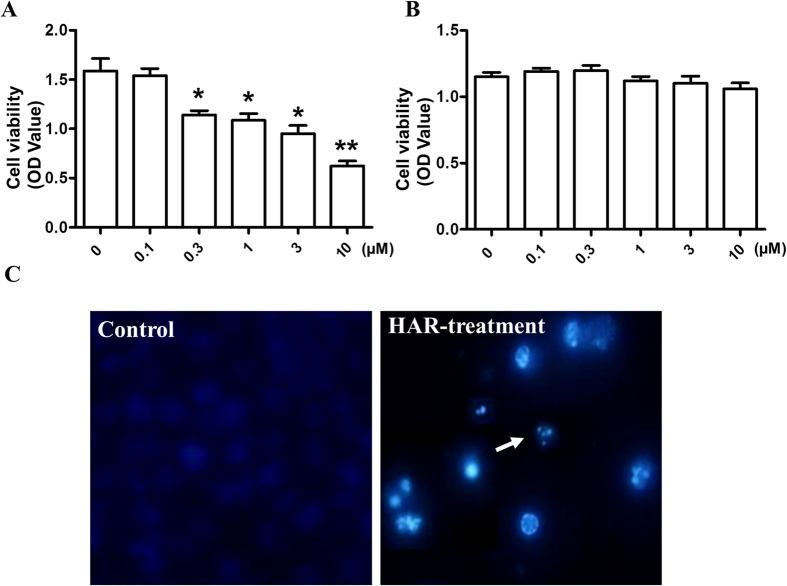
Antiproliferative activity of HAR. (**A**) SGC-7901 cells were treated with different concentrations of HAR (0–10 μM) for 48 h. Cell viability was then measured using MTT method. (**B**) 3T3 cells were treated with different concentrations of HAR (0–10 μM) for 48 h. Cell viability was then measured using MTT method and the optical density (OD) value was determined at 570 nm. (**C**) The cells were treated without or with 5 μM HAR for 48 h, then the cells were stained by Hoechst 33258, the nuclear morphology of the cells was observed under a fluorescence microscope. Data are presented as means ± SD of three independent tests. *p < 0.05 versus control, **p < 0.01 versus control.

To determine the effect of HAR on tumor cell proliferation, SGC-7901 cells were treated with various concentrations of HAR for 48 h and cell viability was determined using MTT assay. Proliferation of SGC-7901 cells was markedly inhibited by HAR-treatment in a dose dependent manner with a lower IC_50_ value of 4.08 ± 0.89 μM (Fig. 2A) and no obvious cytotoxicity was observed in mouse fibroblast cells (3T3) (Fig. 2B). To further determine the characteristics of HAR-induced SGC-7901 cell death, morphologic change was observed. In SGC-7901 cells, exposure to HAR for 48 h resulted in morphologic characteristic alterations of apoptosis, including membrane blebbing, nuclear condensation and granular apoptotic bodies (Fig. 2C) compared with the control group.

### HAR induced G2/M cell cycle arrest in SGC-7901 cells

There were some reports that β-carbolines were specific inhibitors of Cyclin dependent kinases. To further investigate the functional mechanism of HAR in inhibiting cell growth, the effect of HAR on the regulation of the cell cycle in SGC-7901 cells was investigated.

**Figure 3:**
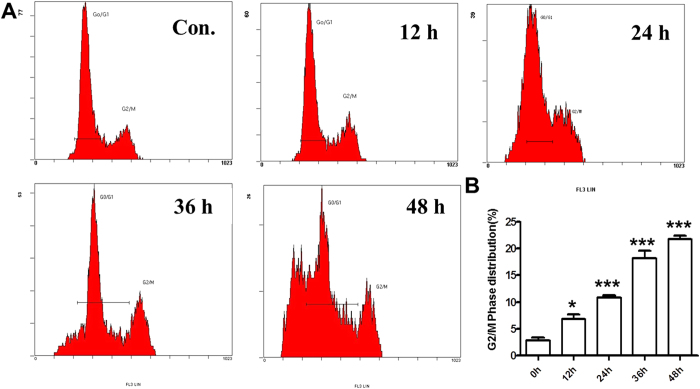
Effect of HAR on cell cycle distribution in SGC-7901 cells. Cell cycle distribution was monitored by Flow cytometry. (**A**) PI staining assay was performed after 5 μM HAR-treatment for the indicated time periods. (**B**) Statistical analysis of G2/M phase distribution. Data are presented as means ± SD of three independent tests. *p < 0.05 *versus* control, ***p < 0.001 *versus* control.

Flow cytometry analysis was performed on the SGC-7901 cells treated with or without HAR for different time points. Interestingly, the results clearly showed that HAR-treated SGC-7901 cells were arrested in G2/M phase in a time-dependent manner (Fig. 3). After 48 hours of incubation, HAR arrested SGC-7901 cells at the G2/M phases (21.1% ± 2.04%) as compared to the control (2.2% ± %1.72%), and also the sub-G1 peak was observed by FACS analysis due to the DNA fragmentation in apoptotic cells.

### Effects of HAR on the expressions of cell cycle regulatory proteins

**Figure 4:**
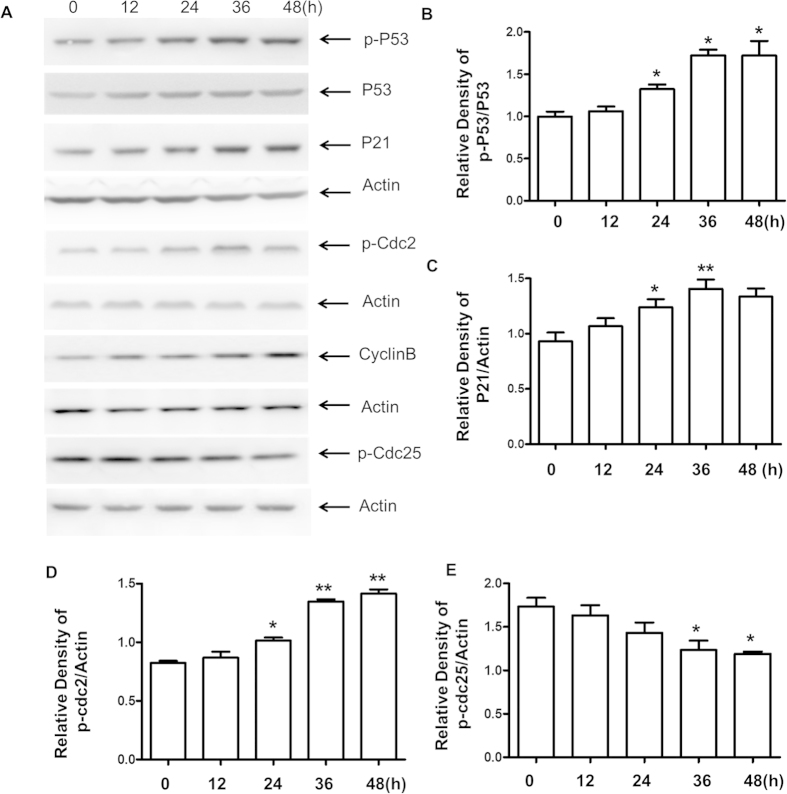
Effects of HAR on cell cycle regulatory proteins in SGC-7901 cells. (**A**) The levels of cell cycle-related proteins including p21, p-P53, P53, Cdc25C, cyclin B1 and p-Cdc2 were assessed by western blot analysis. Twenty micrograms of total protein from each sample were loading for each channel. Actin was used as an internal control from the same membrane. (**B–E**) Statistical analysis of cell cycle arrest related-proteins. Data are presented as means ± SD of three independent tests. *p < 0.05 versus control, **p < 0.01 versus control.

To further characterize the mechanism by which HAR induced G2/M cell cycle arrest, the effects of HAR on the expressions of P21, p-P53, P53, Cyclin B1, p-Cdc2 and p-Cdc25C were examined. As expected, the cellular level of P21, p-P53 and Cyclin B1 dramatically increased after 24 h HAR-treatment and continued to increase up to 48 h. However, HAR-treatment resulted in a remarkably time-dependent decrease in p-Cdc25C expression levels accompanied the positive augment of p-Cdc2 (Fig. 4A). These data strongly indicated that HAR might induce G2/M arrest by altering the expressions of cell cycle related proteins (Fig. 4B–E).

### Effects of HAR on the expressions of extrinsic apoptotic related proteins

**Figure 5:**
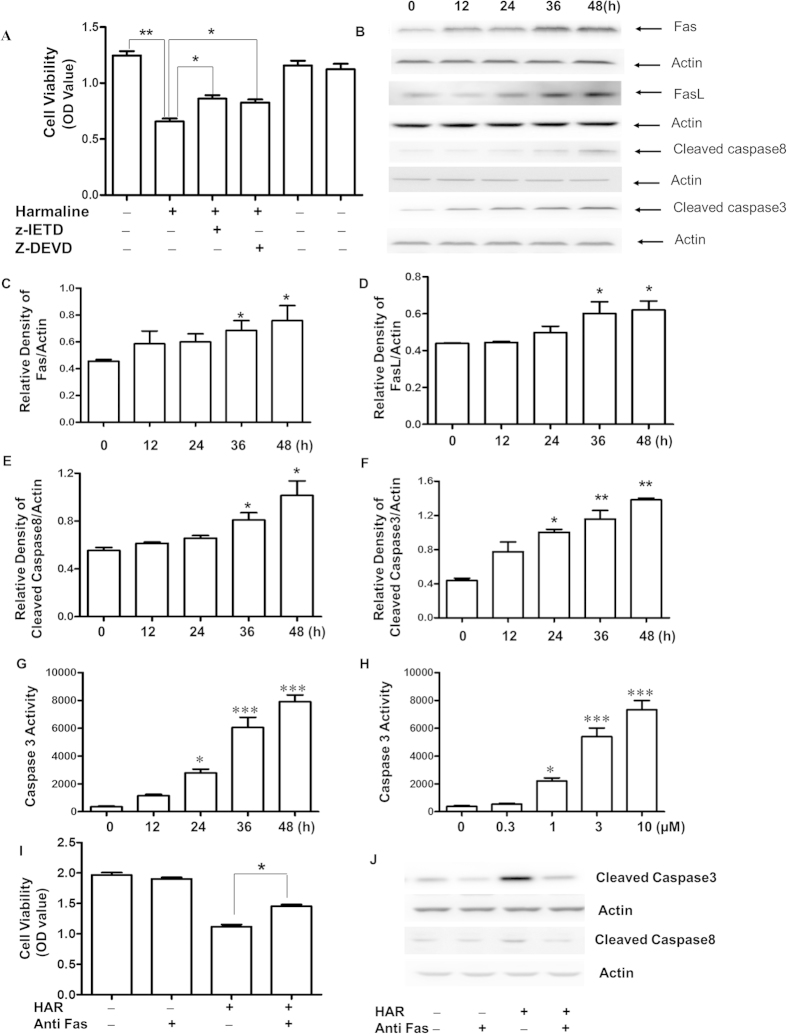
Effects of HAR on apoptotic-related protein and Caspase-3 activity in SGC-7901 cells. Levels of Fas/FasL, activated Caspase-8 and Caspase-3 were assessed by western blot analysis. (**A**) The cells were pretreated without or with z-DEVD-fmk (20 μM) or z-IETD-fmk(20 μM), and then treated with 5 μM Harmaline for another 48 h and the cell viability was measured by MTT assay and the optical density (OD) value was determined at 570 nm. n = 3. (**B**) The cells were treated with 5 μM of Harmaline for the indicated time periods. After treatment, the expressions Fas/FasL, activated Caspase-8 and Caspase-3 were assessed by western blot analysis. Actin was used as an internal control from the same membrane. Statistical analysis of Fas/FasL and activated Caspase-8 and Caspase-3 (**C–F**). (**G–H**) Analysis of Caspase-3 activity. SGC-7901 cells were treated with HAR as indicated. Data are presented as mean ± SD of three independent experiments. *p < 0.05 versus control, **p < 0.01 versus control and ***p < 0.001 versus control. **(I)** The cells were pretreated without or with 1 μM anti Fas antibody for 2 h before 5 μM HAR treatment for another 48 h and the cell viability was measured by MTT assay and the optical density (OD) value was determined at 570 nm. n = 3. **(J)** Anti-Fas antibody prevented caspase-8 and caspase-3 activation by HAR. Caspase-3 and caspase-8 activation were determined by the Western blot. The cells were treated the same as **(I).**

Since HAR showed the higher sensitivity and selectivity on SGC-7901 cells than other cell lines based on our previous study, the main apoptosis-related protein levels were further evaluated. Interestingly, it found that both z-DEVD-fmk (Caspase-3 inhibitor) and z-IETD-fmk (Caspase-8 inhibitor) increased the cell viability compared with cells treated by HAR only (Fig. 5A). It also found that the expressions of Fas/FasL were up-regulated after HAR-treatment accompanied by the positive augments of the activated Caspase-8 and Caspase-3 (Fig. 5B–F). Moreover, Caspase-3 activity assay was applied to determine whether Caspase-3 involved in the HAR-induced apoptosis. As presented in Fig. 5G,H, the activity of Caspase-3 was significantly increased after HAR treatment in a time and dose dependent manner. To further confirm the death receptor (extrinsic) apoptotic pathway contributed to cell death treated by HAR, the additional experiments were carried out. Blocking Fas/FasL signaling using an anti-Fas antibody markedly inhibited the cell death caused by HAR (Fig. 5I). In addition, activated Caspase-3 and Caspase-8 were also downregulated after blocking Fas/FasL signaling (Fig. 5J). These experiments demonstrated that the Fas death receptor pathway contributed to HAR-induced apoptosis.

### Antitumor activity and toxicity of HAR *in vivo*

**Figure 6:**
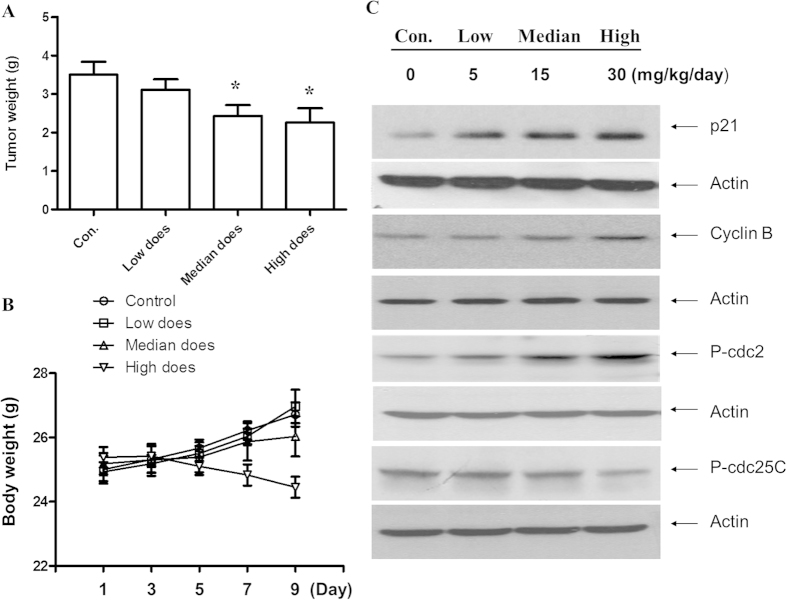
HAR prevented the subcutaneous xenograft tumor growth *in vivo*. (**A**) The average tumor weights in HAR treatment group and control group were measured at the end of the experimental day (10 days treatment). *p < 0.05, n = 10. (**B**) The toxicity of HAR in SGC-7901 xenograft mouse model. Body weights of all of mice were assessed every other day after HAR treatment. (**C**) Expressions of cell cycle regulatory proteins on tumor tissue using Western Blot analysis. Fifteen micrograms of total protein from each sample were loading for each channel. Actin was used as an internal control from the same membrane. *p < 0.05 versus control.

Based on the *in vitro* findings, the efficacy of HAR in SGC-7901 cells in nude mice was evaluated and three different doses of HAR (5, 15, 30 mg/kg/day) were studied. Compared with the control group, both median (15 mg/kg/day) and high doses (30 mg/kg/day) could significantly inhibit the growth of human gastric tumors in nude mice in a dose dependent manner (P < 0.001) (Fig. 6A). Meanwhile, body weight loss was observed after high dose treatment. Based on the therapeutic efficacy of HAR *in vivo* model, the expressions of p21, p-Cdc2 and p-Cdc25C were examined in the tumor samples. As shown in Fig. 6B, HAR treatment significantly increased the expressions of p21, p-Cdc2 accompanying by the reduction of p-Cdc25C. These results strongly suggested that HAR might induce apoptosis through cell cycle arrest *in vivo*. Regarding the toxicity in high dose treatment, the adverse effects, particularly in liver, were examined. The liver enzymes AST and ALT in serum level were normally used as biomarkers of hepatotoxicity. In this study, no alteration of the ALT or AST in serum level was observed, except high dose group. Moreover, the contents of serum WBC, RBC, HGB and PLT also had no change in the low and median dose treatment group (Table 1). Therefore, the modest HAR-treatment without induced apparent systemic toxicity, and strengthened its use for gastric tumor. However, the high dose treatment could result in the WBC reduction and enhancing the level of ALT.

## Discussions

Gastric cancer is a leading cause of death worldwide. Although surgery is the mainstay of any curative treatment, recurrences and metastases are still observed in approximately two-thirds of patients^18^. HAR is the one of major alkaloids in *P. harmala* seeds which exerts main function. In the present study, we demonstrated a novel molecular mechanism through which HAR inhibited gastric cancer cell proliferation *in vitro* and *in vivo*.

HAR showed a significantly inhibition in cell proliferation of SGC-7901 cells in a dose dependent manner. Also SGC-7901 cells underwent apoptosis after HAR treatment based on the typical morphologic change and sub-G1 fraction resulting from DNA fragmentation. Identification of the sub-G1 subpopulation may be used as an index of apoptotic cells in SGC-7901 cells treated by HAR.

Moreover, the results showed that HAR exerted a strong anti-proliferative effect against gastric cancer cells by inducing G2/M arrest. As we know, cell cycle progression is regulated by Cyclin-dependent kinases (Cdks) and Cyclins. Cyclin B-Cdc2 plays a main role in G2/M phase transition. Cyclin dependent kinase inhibitor (CDKI), Cip1/p21, also regulates the transitions of G2/M phase by binding and inhibiting the activity of Cdc2-Cyclin B1 complex. The ability of p21 to promote cell cycle inhibition may also depend on its ability to mediate p53-dependent gene repression, as p21 is both necessary and sufficient for p53-dependent repression of genes regulating cell cycle progression^19^,^20^. Cdc25C is another critical regulator of Cdc2-Cyclin B1 kinase activity and control cell cycle progression by dephosphorylating and activating CDKs^21^,^22^,^23^. Consistent with these notions, we observed an increase in the protein level of inhibitory p21 in SGC-7901 cell. An increased phosphorylation of Cdc2 accompanied by a decreased p-Cdc25C protein was also observed in SGC-7901 cells treated with HAR. The increased Cdc2 phosphorylation was likely due to reduced p-Cdc25C protein level which prevented it from dephosphorylating Cdc2.

**Figure 7:**
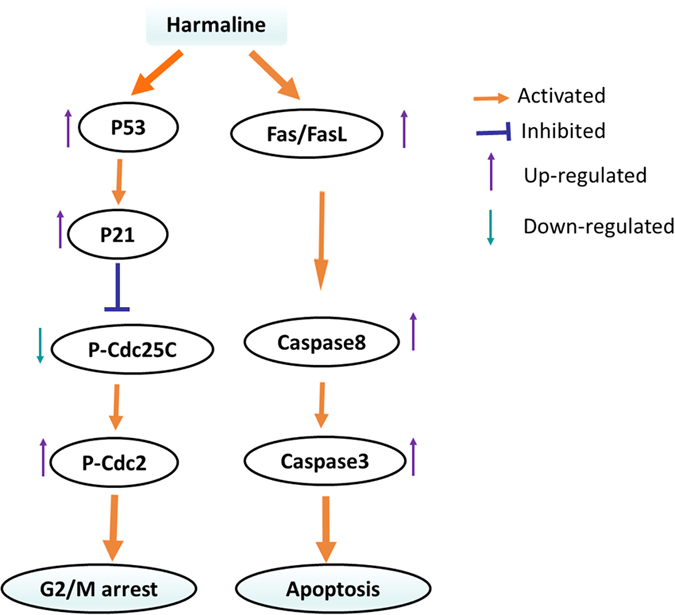
Proposed mechanisms of HAR-induced G2/M phase arrest and apoptosis in SGC-7901 cells.

Fas/FasL death receptor plays an important role in cell apoptosis. Upon binding to the FasL, the Fas trimerizes and induces apoptosis through the cytoplasmic death domain (DD) that interacts with signaling adaptors like Fas-associated death domain (FADD). FADD carries a death effector domain (DED) and it recruits the DED containing procaspase-8 protein which is in inactive state. Procaspase-8 is proteolytically activated to Caspase-8. FADD also helps in the activation of Caspase-10. Upon activation, Caspase-8 and Caspase-10 cleave and activate downstream effector Caspases, including Caspase-3, 6 and 7. But there was also reported that harmol activated a key element of the Fas signaling pathway independently of Fas/FADD activation^24^. Therefore, Fas, FasL, and two key downstream effector Caspases, activated Caspase-8 and Caspase-3 were detected to reveal whether Fas/FasL signaling pathway involved in inducing apoptosis of SGC-7901 cells upon treatment of HAR. Surprised, up-regulation of Fas, FasL, activated Caspase-8 and caspase-3 was observed in SGC-7901 cells. The Caspase-3 activity was also detected in gastric tumor cells. As expected, increased Caspase-3 activity was also observed in a time or dose dependent manner. Blocking Fas/FasL signaling using an anti-Fas antibody significantly blocked Caspase-3 activation, and Caspase-8 activation. These results implied that Fas/FasL signaling pathway was initiated and involved in SGC-7901 cell apoptosis (Fig. 7). However, it was not known if intrinsic apoptotic pathway also contributed to SGC-7901 cells apoptosis treated by HAR. It was still needed to be verified further in the future.

The strong tumor inhibition properties, as well as the Fas/FasL-mediated apoptotic action of HAR, prompted us to evaluate its efficacy and safety *in vivo*. In our current experiments, HAR suppressed tumor growth at a dose of 15 mg/kg/day without any significant changes of the mice body weight and the possible mechanism was cell cycle arrest owning to the up-regulating P21 and p-Cdc2. Side effects, such as hair loss, lethargy, dysphoria, or other macroscopical visceral pathogenic changes were not observed. However, it was reported that HAR could significantly reduced the viability of four human cultured non-transformed (CCD18Lu) and transformed (HeLa, C33A and SW480) cells in a dose-dependent manner^25^. In this study, only high does of HAR (30 mg/kg/day) treatment showed toxicity based on the body weight loss, the serum WBC reduction and the increasing of ALT level. Therefore, enhanced *in vivo* studies and narrow research was still required to draw a line between cytotoxic and antitumor efficiency of HAR.

Taken together, the induction of P21 at early stage, along with the increased p-Cdc2 as a result of the downregulation of Cdc25c, lies at the base of the mechanism through which HAR induced G2/M arrest and the activation Fas/FasL pathway (Fig. 7).

## Methods

### Materials

HAR was isolated and identified in the Laboratory of Natural Lead Compound of Guangdong Pharmaceutical University. p-Cdc2 and p-Cdc25C were purchased from Santa Cruz Biotechnology (Santa Cruz, CA, USA). Rabbit polyclonal anti-p21, p-P53, P53, Cyclin B, Caspase-8, Caspase-3, antibody against FasL, Fas and FasL were purchased from Cell Signaling Technology (Beverly, MA, USA). Hoechst 33258 were purchased from Beyotime Institute of Biotechnology (Suzhou, China). Cell cycle detection kit was purchased from BD Bioscience (San Diego, CA, USA). MTT were purchased from Sigma-Aldrich Chemical Co. (St. Louis, MO, USA). Z-Ile-Glu-Thr-Asp-FMK (Z-IETD-FMK) and Z-Asp (O-Me)-Glu(O-Me)-Val-Asp(O-Me) fluoromethyl ketone (Z-DEVD-FMK) were purchased from Calbiochem (Gibbstown, NJ, USA). Penicillin and streptomycin were purchased from Hyclone (Logan, UT, USA). Caspase-3 activity kit was purchased from Cell Signaling Technology (Danvers, MA, USA). Flow Cytometer was purchased from BD Bioscience (San Diego, CA, USA). Microplate reader was purchased from Tecan (TECAN, Switzerland). HF-120 automatic biochemical analyzer was purchased from Healife (Shangdong, China).

### Extraction and purification of HAR from *P. harmala*

The dried seeds of *P. harmala* (11 kg) were powdered and then extracted three times with 70% aqueous methanol under reflux. After filtration and evaporation of the solvent under reduced pressure, the residues (2.3 kg) were suspended in water and acidified to pH 1.0 with 10% hydrochloric acid. Lipophilic impurities were removed with CHCl_3_ extraction and the aqueous fraction was alkalized to pH 7.0 with concentrated NH_3_∙H_2_O. The crude alkaloids were extracted four times with CHCl_3_ and n-BuOH sequentially. The CHCl_3_ fraction (200 g) was separated into 15 fractions (C1–C15) by silica gel column chromatograph (200–300 mesh, 100 × 1700 mm) eluted with CHCl_3_/MeOH/Triethylamine (TEA) (100:0:0.1 to 0:100:0.1, v/v/v), in increasing order of polarity. Fraction C12 (29.4 g) eluted by CHCl_3_/MeOH/TEA (20:1:0.1) was subjected to further separation by silica gel column (200–300 mesh, 60 × 1100 mm) with CH_2_Cl_2_/MeOH (100:0 to 0:100) successively to yield 9 fractions (C12-1–C12-9). Subfraction C12-7 was then chromatographed on an Al_2_O_3_ column (20 × 1100 mm) eluted with CHCl_3_/MeOH (100:0 to 0:100) to give a yellow needle-shaped crystal in C12-7-2 (400 mg). A purified compound was obtained by recrystal1ization from chloroform-methanol (1:1).

### Cell culture

Human gastric carcinoma SGC-7901 and mouse fibroblast 3T3 cell lines were purchased from American Type Culture Collection (ATCC) (Manassas, VA, USA). Cells were cultured in Dulbecco's Modified Eagle's Medium (DMEM) supplemented with 10% fetal bovine serum (FBS), 100 U/mL penicillin and 100 μg/mL streptomycin and maintained in a humidified atmosphere containing 5% CO_2_ at 37 °C. HAR was dissolved in methanol and diluted by DMEM keeping a maximum concentration of methanol 0.1% in the culture medium.

### Growth-inhibitory effect of HAR in SGC-7901 cell line

MTT method was utilized to evaluate the bioactivity of HAR. Briefly, SGC-7901 cells or 3T3 cells were cultured in DMEM with 10% FBS and maintained at 37 °C in 5% CO_2_. A number of 2.5 × 10^4^ cells were seeded in each well of a 96-well plate before drug treatment. After overnight incubation, the cells were treated by with HAR either at 0, 0.1, 0.3, 1, 3, and 10 μM for 48 h or the SGC-7901 cells were pretreated with or without z-DEVD-fmk (20 μM), z-IETD-fmk (20 μM) for 1 h, then treated with 5 μM HAR for an additional 48 h. For Fas/FasL blocking assay, SGC-7901 cells were pretreated with or without a neutralizing antibody against Fas (1 μM) for 2 h and then treated with 5 μM HAR for an additional 48 h. Cell viability was determined by the MTT assay. Experiments were performed in triplicate for each sample. Data were presented as mean ± SD of three independent experiments.

### Detection of morphological apoptosis with Hoechst 33258 staining

SGC-7901 cells in DMEM containing 10% FBS were seeded into 24-well plates with 1 × 10^4^ cells for each well and incubated overnight. 5 μM HAR was added to 24-well plates and incubated for another 48 h. After incubation, the supernatant was removed gently and the cells were washed by ice-cold PBS. Finally, the cells were fixed with 3.7% paraformaldelyde at room temperature for 30 min, then washed twice with Ca^2+^ and Mg^2+^ free PBS and stained with 50 μM Hoechst 33258 for 15 min at 37 °C in the dark. After incubation, the nuclear morphology of the cells was observed under a fluorescence microscope.

### Flow cytometry cell cycle propidium iodide (PI) assay

SGC-7901 cells were stained by PI according to the manufacturer’s protocol. Briefly, SGC-7901 cells in DMEM containing 10% FBS were seeded into 10 cm dish with 2 × 10^5^ cells for each dish and incubated overnight. Then the cells were treated with 5 μM HAR for 0, 12, 24, 36 or 48 h. After incubation, a suspension of cells with a density of 10^5^ cells per treatment was fixed in 3.7% ice-cold paraformaldehyde for 30 min, followed by labeling with PI. The labeled cells were analyzed using a Flow Cytometer.

### Western Blot analysis

SGC-7901 cells were washed with cold PBS after 5 μM HAR treatment in different time points as indicated, and then the cells were lysated in a RIPA buffer that contained a mixture of protease inhibitors or the solid tumors were cut into small pieces and then were lysated in a RIPA buffer that contained a mixture of protease inhibitors. Twenty micrograms of total protein from each sample were run on a 12% SDS-PAGE gel and transferred to a nitrocellulose membrane. The membranes were incubated with appropriately diluted primary antibodies, followed by horseradish peroxidase-conjugated secondary antibodies against the corresponding species. Labeling was detected using the ECL system (Amersham Biosciences, Pittsburgh, PA, USA).

### Caspase-3 activity assay

Caspase-3 activity assay is a fluorescent assay that detects the activity of Caspase-3 in cell lysates. It contains a fluorogenic substrate (N-Acetyl -Asp-Glu-Val-Asp-7-amino-4-methylcoumarin or Ac-DEVDAMC) special for Caspase-3. SGC-7901 cells were seeded into a 96-well plate before the experimental day and then the cells were treated with 5 μM HAR in different time courses or the cells were treated by different concentrations of HAR for 48 h. After incubation, both floating and adherent treated-cells were then collected and lysated in ice-cold lysis buffer for 10 min in a 1.5 mL tube. After centrifuged for 10 min at 4 °C, the supernatant was transferred to another tube and then mixed 200 μL of substrate solution B and 25 μL lysate solutions in a black plate appropriate for fluorescent assay. The plate was incubated at 37 °C for 1 h in the dark and it was read on a fluorescence plate reader according to the assay protocol. Data were presented as mean ± SD of three independent experiments.

### Mouse experiments and tumor xenograft model

Healthy male nude mice (Balb/c, 6–8 weeks of age) were bought from Guangdong Medical Experimental Animal Center. All animal experiment protocols were approved by the Animal Ethics Committee of Wuhan University. Mice were injected subcutaneously with SGC-7901 cells (1 × 10^7^ cells/mouse) to the right back. After one week, animals were randomly divided into four groups (10 mice for each group). Mice were treated with either 5% CMC-Na or HAR (low dose group: 5 mg/kg/day; median dose group: 15 mg/kg/day; high dose group: 30 mg/kg/day) dissolved in 5% CMC-Na by oral and were weighted every other day. Finally, mice were sacrificed after 10 days treatment. Twenty-four hours before the last treatment, animals were anesthetized after exposure to ether in desiccators kept in a well-functioning hood. Blood samples were collected by heart puncture, and serum samples were tested using Healife automatic biochemical analyzer. The whole solid tumor tissues were detached from the back of mice and then were frozen in liquid nitrogen for Western Blot analysis after weight.

All of the animals were treated according to protocols approved by the Animal Ethics Committee of Wuhan University. And the study was approved by the Animal Ethics Committee of Wuhan University.

### Statistical analysis

Data were presented as mean ± standard deviation from three experiments in three different biological replicates, and the significance was evaluated by one-way analysis of variance (ANOVA).

## Additional Information

**How to cite this article**: Wang, Y. *et al.* Novel mechanism of harmaline on inducing G2/M cell cycle arrest and apoptosis by up-regulating Fas/FasL in SGC-7901 cells. *Sci. Rep.* **5**, 18613; doi: 10.1038/srep18613 (2015).

## Figures and Tables

**Figure 1:**
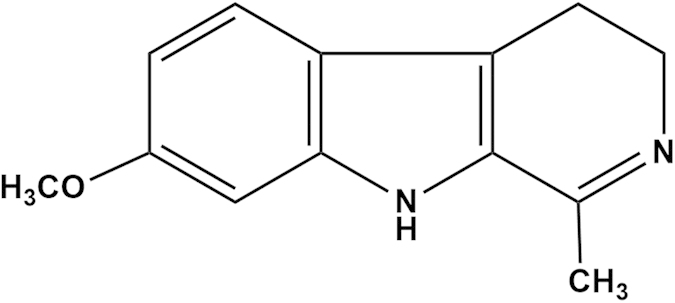
Chemical structure of HAR.

**Table 1:** Effects of Harmaline on haematological parameters and the level of ALT, AST in the serum.

Group	WBC	RBC	HGB	PLT	ALT	AST
(n = 6)	(×10^9^/L)	(×10^9^/L)	(g/L)	(×10^9^/L)	(U/L)	(U/L)
Control	9.4 ± 0.1	9.0 ± 0.1	144.2 ± 8.6	964.2 ± 23.4	53.2 ± 2.2	62.4 ± 2.5
Low does	9.1 ± 0.5	9.1 ± 0.2	147.5 ± 3.6	962.7 ± 11.2	52.2 ± 1.8	60.9 ± 3.8
Median does	8.8 ± 0.5	8.9 ± 0.1	142.0 ± 8.2	948.3 ± 10.4	51.0 ± 1.6	59.0 ± 2.6
High does	8.2 ± 0.8^*^	8.6 ± 0.4	134.0 ± 6.5	913.5 ± 26.0	68.9 ± 9.5^*^	69.7 ± 6.7

Note:1 WBC: white blood cells; RBC: red blood cells; HGB: hemoglobulin; PLT: platelet; AST: aspartate aminotransferase; ALT: alanine aminotransferase.

2 The values are expressed as mean ± SD of six mice per group.

3 *Significantly different from the control group.
